# Cone-Beam Computed Tomography Study of Incisive Canal and Maxillary Central Incisors in Dravidian Population

**DOI:** 10.7759/cureus.63707

**Published:** 2024-07-02

**Authors:** Abirami Selvaraj, Aravind Kumar Subramanian

**Affiliations:** 1 Orthodontics and Dentofacial Orthopedics, Saveetha Dental College and Hospitals, Saveetha Institute of Medical and Technical Sciences, Saveetha University, Chennai, IND

**Keywords:** cone-beam computed tomography (cbct), root resorption, skeletal class ii malocclusion, skeletal class i malocclusion, incisive canal

## Abstract

Introduction: En-masse maxillary anterior retraction is necessary to attain an esthetic profile in Angle's class I bimaxillary dentoalveolar protrusion and Angle's class II division 1 malocclusion. The objective of this study was to evaluate configurational relationships between maxillary incisors and incisive canal in Angle's class I bialveolar protrusion and Angle's class II division 1 malocclusion by cone-beam computed tomography (CBCT).

Methods: A total of 108 adult CBCT scans of 54-skeletal class I bialveolar protrusion and 54-skeletal class II division 1 malocclusions were retrospectively analyzed. Angles between palatal plane and axis of maxillary alveolar border (θ1), incisive canal (θ2), and maxillary right central incisor (θ3) were measured in relation to the midsagittal plane. Linear measurements such as incisive canal width (IC-IC), medial inter-root distance (Rm-Rm), posterior inter-root distance (Rp-Rp), anteroposterior distance from Rm to tangent of right central incisor (11 Rm-Cat), and left central incisor (21 Rm-Cat)_ _corresponding to three vertical levels (L1, L2, and L3) were assessed in axial cross-sectional plane. Association among angular measurements was examined by Spearman correlation coefficient analysis. Mann-Whitney U test compared variables of linear measurements at three vertical levels.

Results: Estimated distance from incisor root to incisive canal was 5-6 mm in both groups slightly influenced by skeletal class and vertical levels but not gender. Mann-Whitney test demonstrated significant differences between groups at three vertical levels (p<0.05). Only θ2 revealed a significant difference (p<0.05) between malocclusions compared to θ1 and θ3. The angular measurements for both malocclusions were positively correlated (p<0.05).

Conclusion: Sagittal root-canal cortical plate distance varied significantly in both malocclusions (5-6 mm). Inter-root distance (Rp-Rp) was greater than incisive canal width (IC-IC) at all three vertical levels indicating a reduced possibility of canal invasion after maximum retraction at posterior levels.

## Introduction

Maxillary anterior teeth play a significant function in esthetics, phonetics, and mastication [1]. One typical orthodontic irregularity in anterior maxillary region is incisor protrusion. This condition is most often seen in Angle's class I bimaxillary dentoalveolar protrusion and Angle's class II division 1 malocclusion [2]. Skeletal class II division 1 malocclusions and bimaxillary protrusion are prevalent among the Dravidian population. Bimaxillary protrusion is due to maxillary and mandibular dentoalveolar flaring and proclined upper incisors which causes lip protrusion and convex facial profile. Notably, adults have alternative interventions involving dental camouflage, orthognathic surgery, and fixed functional appliances [3]. According to patients' perception, the most commonly preferred option is dental camouflage which includes premolar extraction and then maximal retraction of upper front teeth [4,5]. The degree of sagittal disparity in class II raises the difficulty of treatment, especially if it coexists with increasing vertical maxillary excess. Hence, appropriate invasive mechanics is required in addition to retraction. The hard tissues such as cortical plate of maxillary bone and incisive canal are the determining components for tooth movement.

The "envelope of discrepancy" suggested by Ackerman et al., illustrates the limitations of the range of orthodontic, orthopedic, and orthognathic movement of maxillary incisors [6]. However, skeletal anchorage systems such as mini-plates, micro/mini-implants have now broadened the scope of orthodontic tooth movements [7]. Previous researches have shown that anatomical limitations such as a cortical plate of incisive canal, labial, and palatal bone may enhance the likelihood of root resorption in the maxillary anteriors following greater incisor retraction [8]. Some studies have reported that root proximity of maxillary incisors to the incisive canal may influence the degree of root resorption following marked incisor retraction assessed with cone beam computed tomography (CBCT) [9-12]. Due to overlapping facial features, two-dimensional radiographs are inadequate for evaluating the relationship between incisive canal and incisors. Three-dimensional (3D) scans may collate more comprehensive data. The proximity of the anatomical relationship between the maxillary incisors and the incisive canal has been investigated in CT and CBCT studies, but they did not examine in relation to specific factors such as skeletal malocclusions, sex, and specific ethnicity [13-15]. Therefore, the rationale of this research is to examine the association in relation to these specific factors. The novel component of this research would be to find some predictive measures and correlations between incisive canal and maxillary incisor roots for both malocclusions, with an emphasis on the Dravidian population and potential ramifications. Therefore, the aim of the study was to evaluate three-dimensional anatomical relationships between maxillary incisors and the incisive canal in the maxillary alveolar bone between skeletal class I bialveolar protrusion and skeletal class II division 1 malocclusions.

## Materials and methods

This retrospective study involved cone-beam computed tomography (CBCT) images of patients aged between 18 and 35 years, who had reported to the Department of Orthodontics and Dentofacial Orthopedics from December 2013 to February 2022 at Saveetha Dental College and Hospitals for treatment. The study design was approved by the Institutional Human Ethical Committee (IHEC), Saveetha Institute of Medical and Technical Sciences (SIMATS) University with IHEC number: IHEC/SDC/ORTHO-1907/22/423. The study's sample size was estimated using Matsumura et al. [16]. CBCT images of the Dravidian population, aged between 18 and 35 years, Sharp well-contrasted images with upper central incisors without any radiological pathology were included. Patients with missing or supernumerary upper incisors, any trauma, nasopalatine pathology, underwent orthodontic treatment, skeletal asymmetries, and any systemic diseases that hinder bone growth, such as osteogenesis imperfecta, growth hormonal disorders, etc., were excluded.

Analysis of images

Of 350, 145 images were selected which satisfied the selection criteria and segregated into skeletal class I and class II malocclusions by two investigators (AS and AK). Segregation was done based on sella-nasion-A point (SNA), sella-nasion-B point (SNB), and A point-nasion-B point (ANB) angle parameters of Steiner's cephalometric analysis [17]. Thirty-seven CBCT images were excluded due to low quality of scans and image distortions. A final total of 108 CBCT images were divided into the following two groups: group 1, skeletal class I bimaxillary protrusion (54), and group II, skeletal class II division 1 malocclusion (54). All CBCT records were obtained using the CareStream CS 9600 scanner (Rochester, NY: Carestream Dental) with the following parameters: 16x17 field of view cm, 120 kVp, 5 mA, acquisition time: 24 seconds, and voxel size resolution: 150 μm. All CBCT scans were saved in Digital Imaging and Communications in Medicine (DICOM)-3 file format and imported and viewed in Dolphin software (Chatsworth, CA: Patterson Dental). One examiner (AS) recorded all the required measurements. The slice images were randomly assigned numbers, and the examiner blindly re-evaluated the scans in a two-week time interval to check for intra-operator concordance. Any conflict was resolved by discussion between the authors (AS and AK). Inter- and intra-examiner reliability of the measurements was determined to reduce the assessment bias by repeating all the measurements after two weeks for 20% of randomly selected samples.

Standardization and orientation of planes

**Figure 1:**
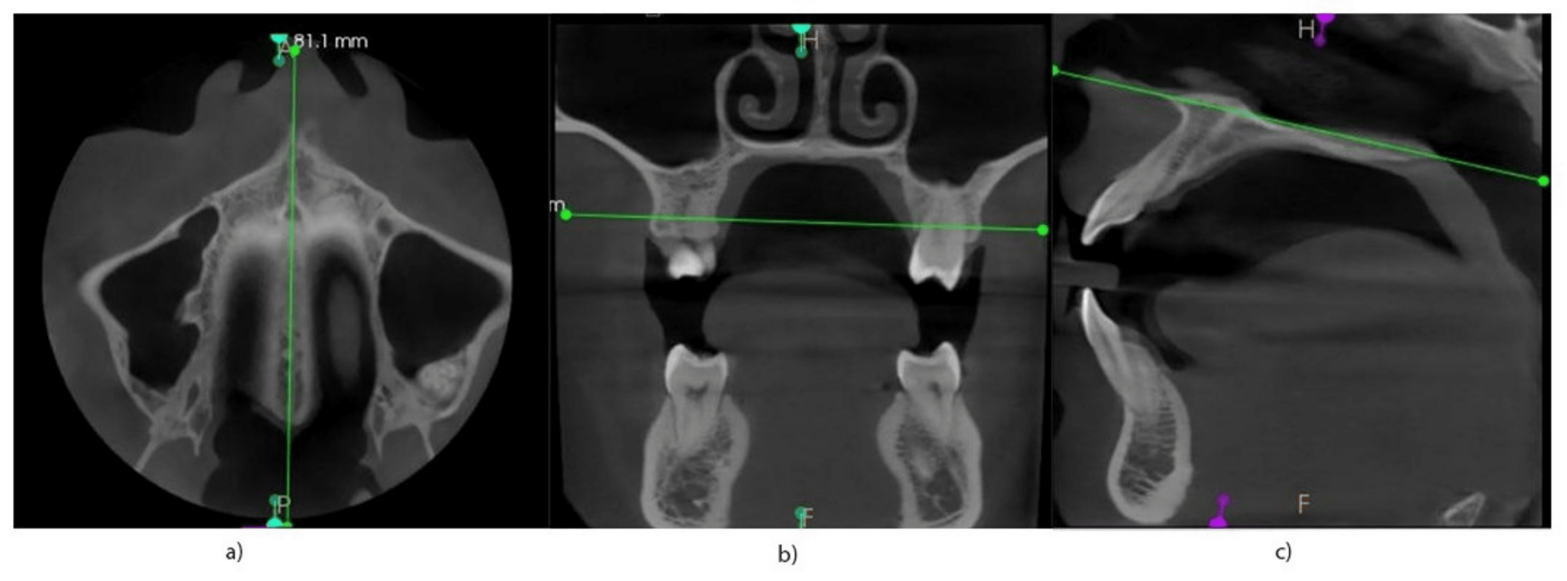
Standard orientation planes and landmarks. The images show (a) sagittal plane, midpalatal suture; (b) coronal plane, passing through greater palatine foramina on two sides; and (c) horizontal plane, palatal plane passing from ANS to PNS. ANS: anterior nasal spine; PNS: posterior nasal spine

All CBCT images were obtained with the head oriented along Frankfort horizontal plane, running parallel to the floor. Prior to measurements, standard orientation planes and landmarks were used (Figures 1a-1c).

**Figure 2:**
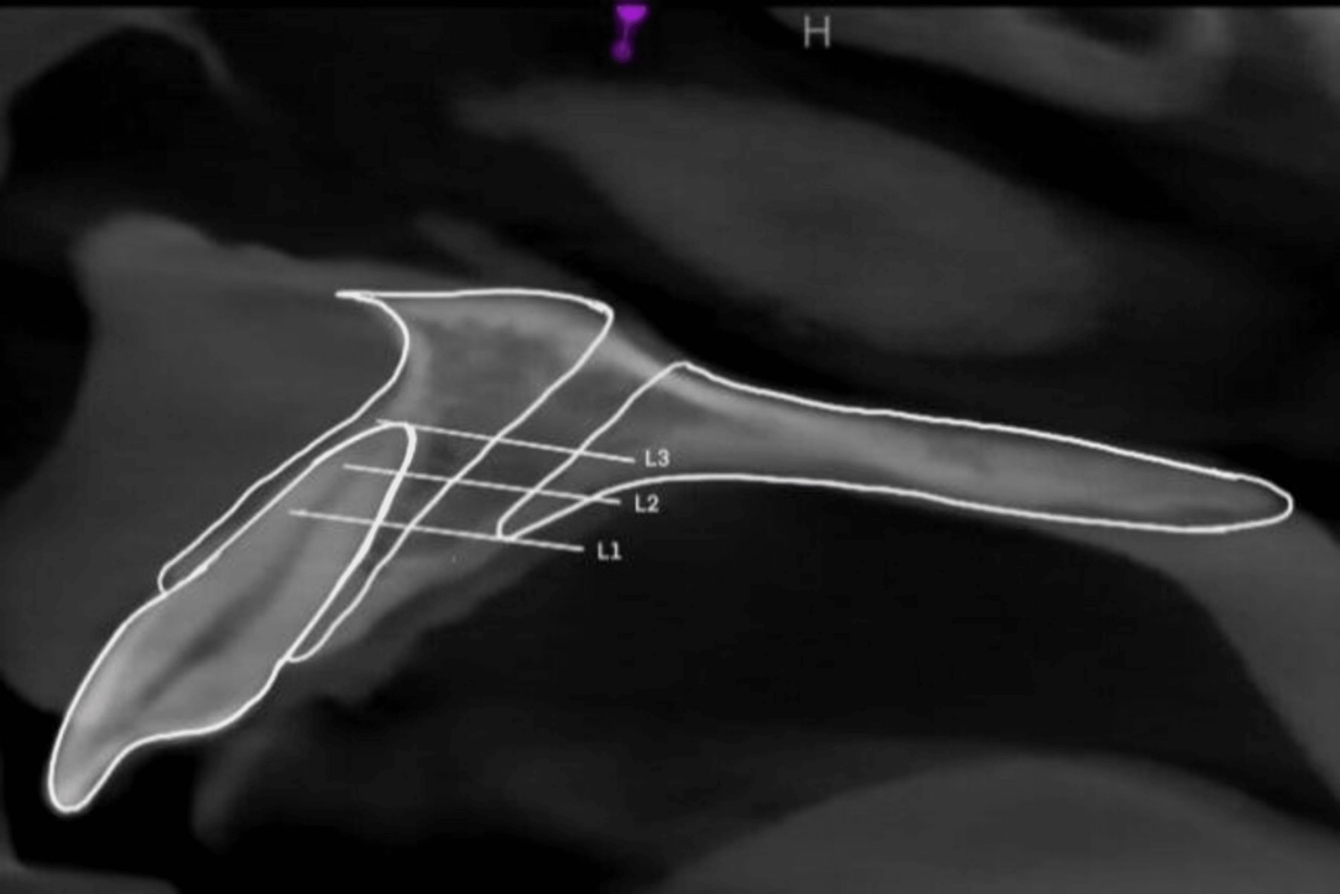
Schematic diagram for three levels: L1, L2, and L3.

Following orientation, all horizontal measurements were taken at three different midsagittal plane levels after orientation as follows: level (L1), oral opening of incisive canal; level (L2), intermediate between L1 and L3; and level (L3), root apex of central incisors (Figure 2).

Parameters assessed at two planes

Midsagittal Plane

**Figure 3:**
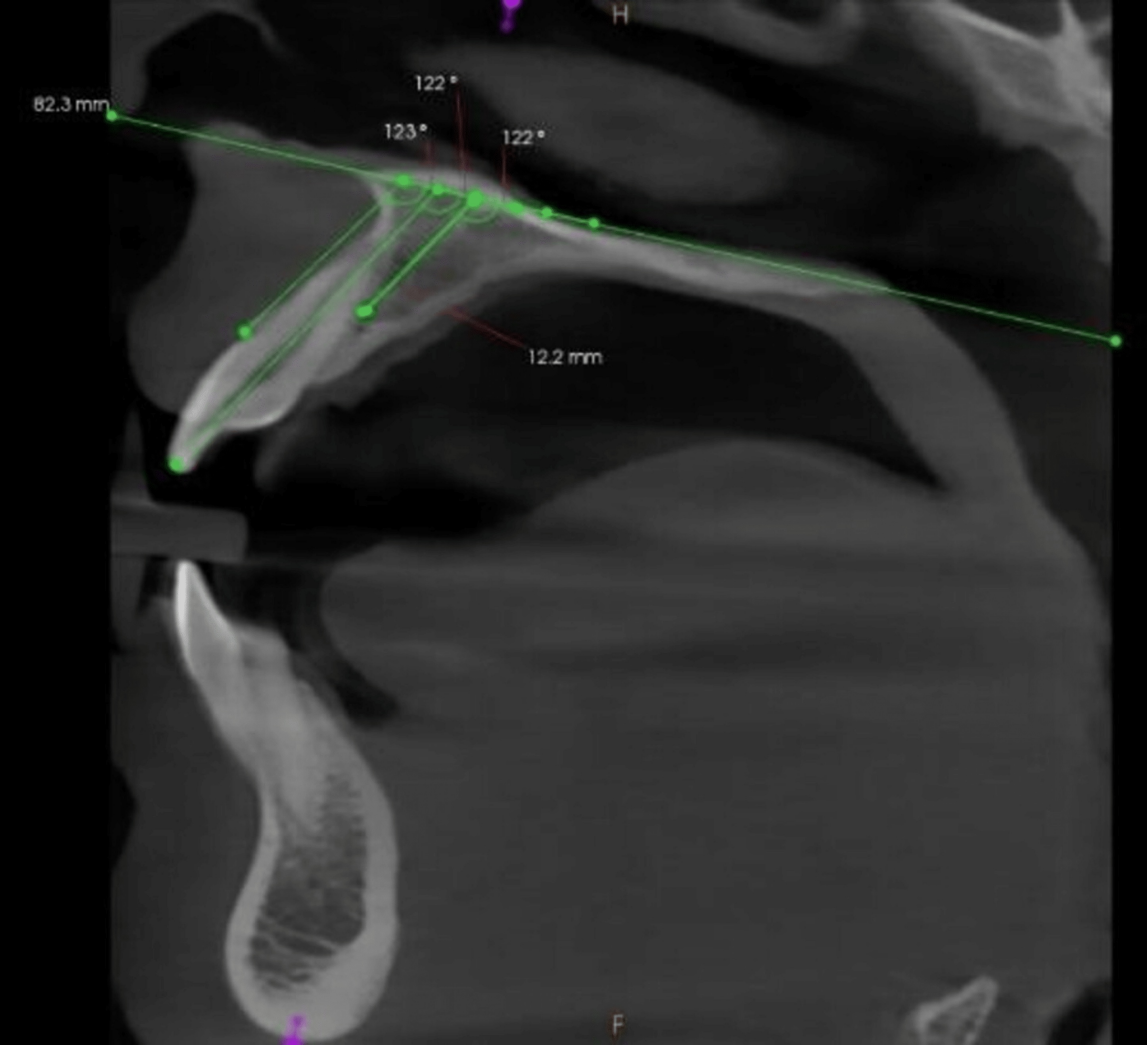
Angles between the palatal plane and axis of the maxillary alveolar border, the incisive canal, and maxillary right central incisor (θ1, θ2, and θ3, respectively).

Angular measurements (θ1, θ2, and θ3) were measured in the midsagittal plane (Figure 3).

Axial Cross-Sectional Plane

**Figure 4:**
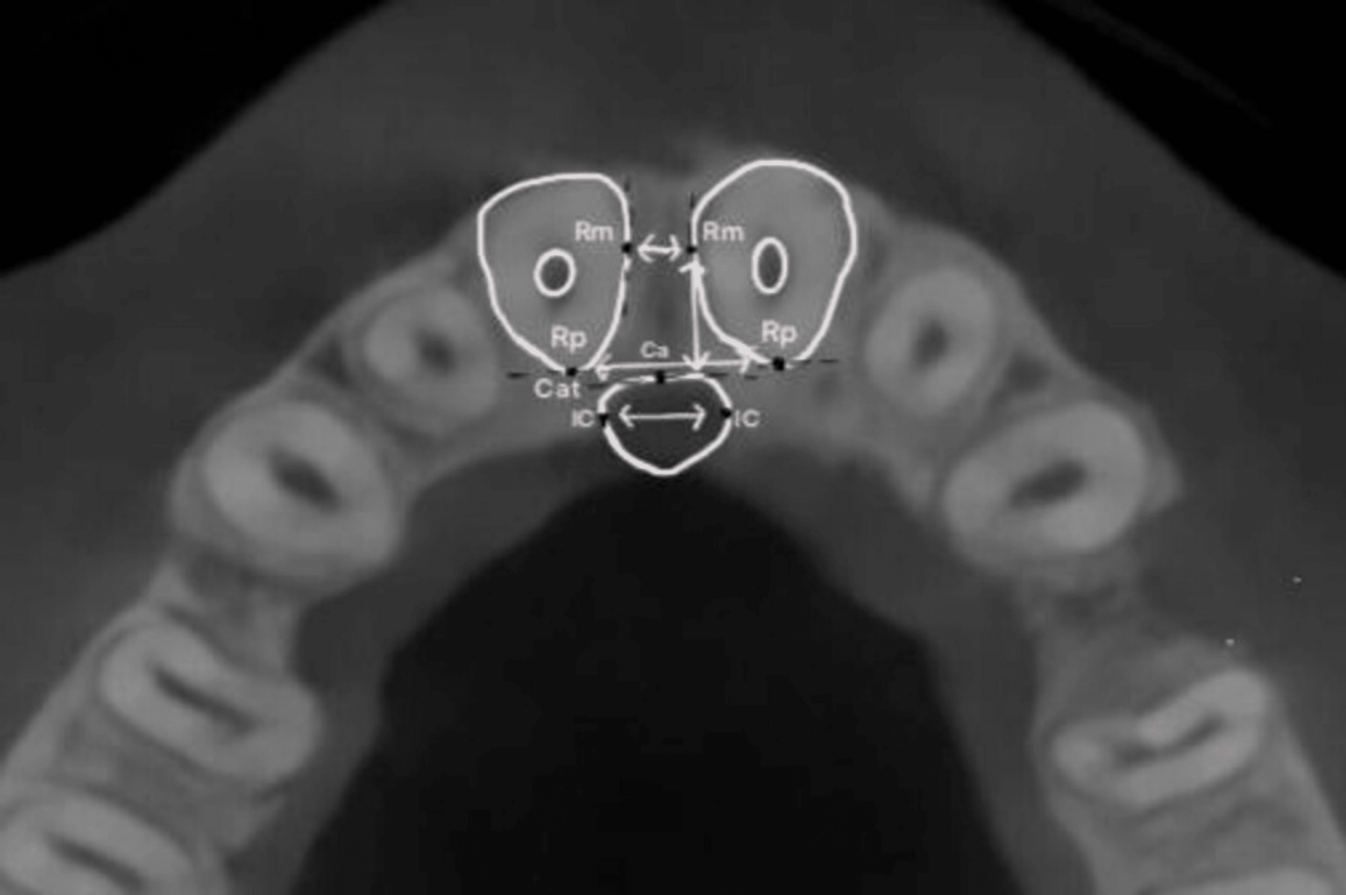
Schematic diagram for linear measurements at axial reconstruction.

Linear measurements (IC-IC, Rm-Rm, Rp-Rp, 11 Rm-Cat, 21 Rm-Cat) were assessed corresponding to three vertical levels that had already been predetermined in the midsagittal plane (Figure 4 and Table 1). 

**Table 1:** Definition of parameters included in the study.

Parameters	Symbols	Description
Linear measurements	IC-IC, Rm-Rm, Rp-Rp, Cat	Incisive canal width, medial inter-root distance, posterior inter-root distance, tangent through Ca
Angular measurements	θ1, θ2, θ3	Angles between palatal plane and axis of maxillary alveolar border, the incisive canal, maxillary right central incisor, respectively

Statistical analysis

Dependent variables failed the Shapiro-Wilk test for normality (p<0.05). This suggests that the assumption of normality for the dependent variables in the dataset has been violated. Mann-Whitney U test was employed to assess linear and angular measures in the midsagittal plane. Association among angular measurements was examined by Spearman correlation coefficient analysis. Inter- and intra-examiner reliability for CBCT scan interpretation was assessed using Cohen's kappa test. Intra-operator concordances were evaluated on the basis of intra-class correlation, which was set ≥0.8. In case of any conflict of interest, it was resolved by discussion with the second author (AK).

## Results

Inter- and intra-examiner reliability

Kappa statistics showed an intra-class coefficient (ICC) value of 0.70 (95% CI: 0.67-0.72), which was suggestive of substantial agreement between the values. Kappa value is 0.934 for inter-examiner reliability and it signifies that the strength of agreement is “very good.” This further underlines the strong inter-operator reliability in this study.

Midsagittal plane

When comparing θ1 and θ3, only θ2 revealed a statistically significant difference (p<0.05) (Table 2). A high positive association was found using the Spearman correlation coefficient between groups I and II (Table 2).

**Table 2:** Spearman correlation coefficients between class I and class II division 1. *P-value <0.05 considered significant. θ1: angle between palatal plane and axis of maxillary alveolar border; θ2: angle between palatal plane and incisive canal; θ3: angle between palatal plane and maxillary right central incisor

Group	Comparisons	Correlation (r)	Coefficient (p-Value)
Class I	θ1 vs θ2	0.255	0.001*
	θ2 vs θ3	0.285	0.001*
	θ3 vs θ1	0.243	0.002*
Class II division 1	θ1 vs θ2	0.290	0.001*
	θ2 vs θ3	0.302	0.001*
	θ3 vs θ1	0.289	0.003*

Axial cross-sectional plane

Mann-Whitney U test showed that both malocclusions differed at all three vertical levels (p<0.05) (Table 3).

**Table 3:** Mean and standard deviation (SD) for variables of linear measurements at three vertical levels for both malocclusions. *P-value <0.05 considered significant. IC-IC: incisive canal width; Rm-Rm: medial inter-root distance; Rp-Rp: posterior inter-root distance; 11 Rm-Cat: anteroposterior distance, Rm to Cat of right central incisor; 21 Rm-Cat: anteroposterior distance, Rm to Cat of left central incisor

Variable	Level	Mean±SD; median		p-Value
		Class I	Class II division 1	
IC-IC	L1	5.0±0.3; 5.0	5.3±0.2; 5.3	0.000*
	L2	4.2±0.1; 4.2	4.5±0.2; 4.6	0.000*
	L3	3.4±0.2; 3.5	3.6±0.1; 3.6	0.000*
Rm-Rm	L1	1.6±0.2; 1.7	1.7±0.2; 1.8	0.001*
	L2	2.2±0.1; 2.3	2.6±0.1; 2.6	0.000*
	L3	7.1±0.1; 3.2	7.9±0.1; 3.4	0.000*
Rp-Rp	L1	6.5±0.1; 6.5	7.1±0.2; 7.9	0.000*
	L2	6.9±0.3; 6.4	7.4±0.2; 7.6	0.000*
	L3	7.1±0.1; 7.2	7.9±0.1; 7.4	0.000*
11 Rm-Cat	L1	6.4±0.1; 6.5	6.8±0.1; 6.9	0.000*
	L2	5.7±0.1; 5.7	5.9±0.1; 6.0	0.000*
	L3	5.2±0.1; 5.2	4.7±0.1; 4.8	0.000*
21 Rm-Cat	L1	6.4±0.1; 6.5	6.8±0.1; 6.9	0.000*
	L2	5.7±0.1; 5.7	5.9±0.1; 6.0	0.000*
	L3	5.2±0.1; 5.2	4.7±0.1; 4.8	0.000*

**Figure 5:**
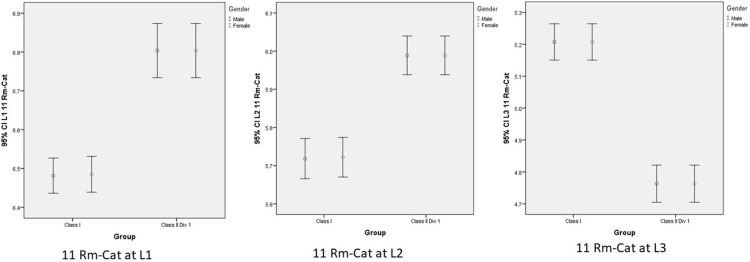
Mean and 95% confidence interval (CI) for the variable of 11 Rm-Cat regarding gender, vertical levels (L1, L2, L3), and skeletal malocclusions. 11 Rm-Cat: anteroposterior distance, Rm to Cat of right central incisor; level (L1): oral opening of incisive canal; level (L2): intermediate between L1 and L3; level (L3): root apex of central incisors

**Figure 6:**
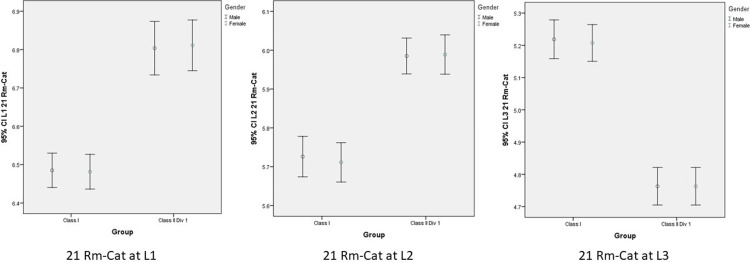
Mean and 95% confidence interval (CI) for the variable of 21 Rm-Cat regarding gender, vertical levels (L1, L2, L3), and skeletal malocclusions. 21 Rm-Cat: anteroposterior distance, Rm to Cat of right central incisor; level (L1): oral opening of incisive canal; level (L2): intermediate between L1 and L3; level (L3): root apex of central incisors

Mean and 95% confidence interval (CI) for the variable of Rm-Ca11 and Rm-Ca21 regarding gender, vertical levels (L1, L2, L3), showed that distance between roots of both maxillary central incisors and incisive canal was slightly influenced by skeletal class and vertical levels but not regarding gender (Figures 5, 6).

## Discussion

The "envelope of discrepancy" about the movement of maxillary incisors has been expanded with temporary anchorage devices (TAD). The maxillary central incisors are most often affected by orthodontically induced inflammatory root resorption, which may be caused by variables relating to the patient or the treatment. The maxillary central incisors are most frequently involved in orthodontically induced inflammatory root resorption which may be associated either with patient-related or treatment-related risk factors. The proximity of incisive canal to maxillary incisor roots peculiar to malocclusion following maximal retraction has not been stated in literature. This may be owing to the use of standard 2D radiography which makes it difficult to evaluate the morphological features of incisive canal. However, 3D radiographs, such as CBCT, have been utilized to identify the anatomical components of incisive canal which may be involved in orthodontically induced inflammatory root resorption of maxillary central incisors during maximal retraction. Hence, the outcomes of this research were acquired to provide morphometric background that would prevent inflammatory root resorption of maxillary central incisors during maximal retraction. To our knowledge, this is the first large-scale CBCT research of relationships between maxillary incisors and incisive canal in the Dravidian population peculiar to malocclusions, such as skeletal class I bimaxillary dentoalveolar protrusion and skeletal class II division 1 malocclusion. Our hypothesis was that ethnicity, types of skeletal malocclusion, and gender may affect the quantitative relationship between the root apex and to incisive canal.

Only θ2 showed a significant difference (p<0.05) between the two malocclusions; θ2 indicates increased proclination of maxillary incisors in group I compared to group II. This may be due to the skeletal class I bony base. This is in accordance with the results of some studies [18,19]. Distance from central incisor roots to incisive canal (11 Rm-Cat, 21 Rm-Cat) demonstrated a significant difference between both malocclusions at all three vertical levels (L1, L2, L3) (p<0.05). This agrees with findings from a research by Costa et al. [14]. Moreover, out of all three vertical levels, distance at L3 is significantly reduced in group II compared to group I. Hence, compared to group I, group II may be more prone to canal proximation after maximal retraction or retraction with intrusion. This may be due to the decreased alveolar bone dimensions in group II compared to group I [20]. Incisive canal width (IC-IC) and inter-root distance at both points (Rm-Rm, Rp-Rp) showed significant increase in group II at all three vertical levels (L1, L2, L3) (p<0.05). This agrees with the findings of some studies [1,21]. The mean value of IC-IC is also quite higher compared to these studies due to anatomical variances.

In this study, we have evaluated inter-root distance in both groups at two points such as most medial and posterior points of roots of central incisors because posterior-median aspect of the apical third of the roots rather than the root apex per se is most likely to approximate with the canal following maxillary anterior retraction and root movements [13,22,23]. When compared to incisive canal width, inter-root distance is smaller at medial points (Rm-Rm) but greater at posterior points (Rp-Rp) in both groups at all three vertical levels (L1, L2, L3). This may indicate that there may be less chance of canal invasion, especially at posterior points of incisor roots in both malocclusions even after maximum retraction. This may be owing to morphological characteristics of central incisor roots and incisive canal. This is in accordance with the results of some studies [9,24]. Furthermore, upon assessing the confounding variables, it was observed that they were impacted by skeletal class and vertical levels, but not by gender. This was corroborated by a study that indicated that gender had no effect on the measured variables [13]. Instead, there exists gender variance [14].

The present study found that anteroposterior distance was approximately 5-6 mm in both groups. Therefore, we can suggest special attention in cases of camouflage for both malocclusions, especially those treated with skeletal anchorage. Skeletally anchored systems help to achieve a greater amount of retraction (7 mm) without losing anchorage but this may also cause root resorption due to the possibility of approximation of incisive canal with palatal cortex [25-27]. For both these malocclusions, orthognathic surgery or corticotomy-assisted retraction would be ideal if anteroposterior distance is less than required to achieve an esthetic profile [28]. The present research suggests that CBCT images may be effective in treating both malocclusions by analyzing incisive canal morphological characteristics to prevent post-operative recession, dehiscence, and root resorption. Along with the diagnosis, force vector and bracket positioning should be considered as incisor root convergence may minimize inter-root distance during retraction.

Limitations

The limitations of this study include specific demographics, retrospective nature, and other malocclusions, as well as growth patterns, that were not investigated. Future investigation is necessary on a larger sample size and would be investigated before executing orthodontic retraction. It would also be interesting to quantify these parameters in growing patients at various time periods since orthodontic therapy is generally begun in children and adolescents.

## Conclusions

Anteroposterior distance between roots of maxillary central incisors and cortical plate of incisive canal showed a significant difference (p<0.05) between both malocclusions, and its mean range was approximately ranging from 5 to 6 mm. Inter-root distance at posterior points (Rp-Rp) is greater than incisive canal width (IC-IC) in both malocclusions at all three vertical levels. This may imply that there may be less chance of canal invasion, especially at posterior points of incisor roots in both malocclusions even after maximum retraction. Moreover, out of all three vertical levels, distance at L3 is significantly reduced in group II than in group I. Hence, group II may be more prone to canal proximation after maximal retraction or retraction with intrusion. 

The results of our study could be clinically helpful in orthodontic treatment of both malocclusions which requires skeletal aided orthodontic retraction of maxillary incisors. Hence, pre-treatment CBCT evaluation should be recommended when a large amount of maxillary incisor retraction and/or intrusion is planned in both malocclusions.
